# Play Equipment, Physical Activity Opportunities, and Children's Activity Levels at Childcare

**DOI:** 10.1155/2012/326520

**Published:** 2012-06-28

**Authors:** Jessica S. Gubbels, Dave H. H. Van Kann, Maria W. J. Jansen

**Affiliations:** ^1^Department of Health Promotion, Maastricht University, P.O. Box 616, 6200 MD Maastricht, The Netherlands; ^2^NUTRIM, School for Nutrition, Toxicology and Metabolism, Maastricht University, P.O. Box 616, 6200 MD Maastricht, The Netherlands; ^3^Academic Collaborative Centre of Public Health Limburg, South Limburg Regional Public Health Service, P.O. Box 2022, 6160 HA Geleen, The Netherlands; ^4^CAPHRI, School of Public Health and Primary Care, Maastricht University, P.O. Box 616, 6200 MD Maastricht, The Netherlands

## Abstract

This study investigated the association between physical activity facilities at childcare (e.g., play equipment) and physical activity of 2- and 3-year olds. Observations of physical activity intensity were performed among 175 children at 9 childcare centers in The Netherlands, using the OSRAC-P. The physical activity facilities were assessed for indoors and outdoors separately, using the EPAO instrument. Regular (single-level) multivariate and multilevel linear regression analyses examined the association of the facilities and child characteristics (age and sex) with children's activity levels. Various physical activity facilities were available in all childcare centers (e.g., balls). Riding toys and a small playing area were associated with lower indoor physical activity levels. Outdoor physical activity levels were positively associated with the availability of portable jumping equipment and the presence of a structured track on the playground. Portable slides, fixed swinging equipment, and sandboxes were negatively associated with outdoor activity levels. In addition, the 3-year old children were more active outdoors than the 2-year olds. In conclusion, not all physical activity facilities at childcare were indeed positively associated with children's activity levels. The current findings provide concrete leads for childcare providers regarding which factors they can improve in the physical environment to facilitate children's physical activity.

## 1. Introduction

 Childhood overweight prevalence is increasing globally, with over 42 million children under 5 years already overweight [1]. Overweight children are at risk for various chronic diseases (e.g., cardiovascular diseases, diabetes) [2] and often remain overweight as adults [3]. The preschool age has specifically been indicated as the most critical growth period for future overweight development [4]. Modifiable determinants of childhood overweight include low level of physical activity [5]. These habits are formed at a young age and often maintained during later life [6, 7]. Targeting physical activity in early childhood is therefore essential to prevent overweight throughout life.

In Europe, over half of the toddlers attend some form of childcare or education facilities [8]. Moreover, various studies have shown an increased overweight risk in children attending childcare [9–11]. The childcare setting offers a potential opportunity for environmental interventions to promote physical activity among young children [12, 13]. Such interventions can only be systematically designed after the most important environmental determinants of physical activity have been identified [14]. 

A review of correlates of preschool children's physical activity level showed that the preschool a child attends is significantly associated with the child's total physical activity [15]. Several authors have called for additional research to identify the specific characteristics of preschools that explain variance in physical activity levels between preschools and childcare centers [16, 17]. However, previous studies mostly summarize or quantify the physical environment into one measure for activity opportunities (e.g., using the Environment and Policy Assessment and Observation (EPAO) instrument or its subscales [18]) or overall quality of the facility (e.g., using the Early Childhood Environment Rating Scale (ECERS-R) [19]), examining the association between such general scores and activity levels [20–26]. Although it is very informative to see whether improving general childcare quality is associated with increased physical activity, it does not provide concrete leads for childcare organizations regarding which factors they can improve in the physical environment to facilitate children's physical activity. The few studies that have previously examined specific characteristics of the physical childcare environment have linked increased physical activity to colour markings at playgrounds [27], but evidence regarding other specific factors such as playground equipment is lacking. The current study therefore examined the association of several specific, separate, physical activity facilities in the physical childcare environment (e.g., play equipment), with the physical activity level of 2- and 3-year olds at childcare.

## 2. Methods

### 2.1. Design and Procedure

The design of the study is based on a study by Bower and colleagues [22]. All nine childcare centers of a large childcare coordinating organization in Maastricht, The Netherlands, were approached to participate in the study. The coordinating organization gave consent to conduct the study and all childcare centers agreed to participate. The childcare centers each catered care for an average of 92 (*sd*⁡ = 28) children, of which an estimated 12% (*sd*⁡ = 14%) were of non-Dutch origin. On average, there were 5 groups (*sd*⁡ = 1) and 20 staff members (*sd*⁡ = 6) per center. Parents were informed about the study in a letter and were able to refuse participation, although none of the parents did so. 

Each childcare centre was visited three times in May and/or June of 2008: once for an interview with the center manager and a rating of the physical activity facilities and twice for direct observations of children's activity level. The observations were performed by two observers, both trained in using the instruments described below. Children were randomly selected for observations and observed simultaneously by both observers. 

The physical activity level was observed by a momentary sampling procedure with observations lasting 15 seconds each. The 30 seconds following the observation period were used to record the observation. This procedure was repeated four times over a period of three minutes for each child. Each child was observed for two nonconsecutive blocks of four observations. In total, 10 children were observed per center per day, resulting in a total of 80 observations. This protocol was implemented on two days for each of the nine centers, during one morning and one afternoon, with at least one week between the two observation days. This resulted in 2880 single observations regarding 180 children (2 observers × 9 centers × 2 days/center × 10 children/day × 2 blocks/child × 4 observations/block).

### 2.2. Instruments and Coding

#### 2.2.1. Physical Activity Levels

During the observations, physical activity level was assessed by means of a translated version of the Observational System for Recording Physical Activity in Children-Preschool Version (OSRAC-P [28]). In line with the OSRAC-P protocol, mean activity intensity during the observation periods (15 seconds) was assessed on a scale from 1 (sedentary) to 5 (highly active). Activity levels ≤2 were regarded as sedentary and activity levels ≥4 as moderate and vigorous physical activity (MVPA [22]).

#### 2.2.2. Physical Activity Facilities

The physical childcare environment (i.e., physical activity facilities) was rated using translated items of the Environment and Policy Assessment and Observation Instrument (EPAO [18, 29]). The EPAO instrument assesses the accessibility of the physical environment, that is, the presence of physical activity facilities such as play equipment. 

Subscales of the EPAO were used, for example, portable and fixed play equipment, and an additional subscale for total size of the playing area. These subscales were rated separately for indoors and outdoors. Portable equipment was rated by checking the availability of 9 types of equipment: balls, climbing structures (e.g., ladders), floor play equipment (e.g., tumbling mats), jumping equipment (e.g., jump ropes, hula hoops), push/pull toys (e.g., wagons), riding toys (e.g., tricycles, cars), slides, sand/water toys (e.g., buckets, scoops), and twirling equipment (e.g., ribbons, batons). Fixed equipment was rated in a similar manner for structured tracks (e.g., playground markings), merry-go-rounds, climbing structures (e.g., jungle gyms), see-saws, slides, tunnels, balancing surfaces (e.g., balance beams), sandboxes, and swinging equipment (e.g., swings, ropes). All play equipment was rated as either present (1) or not (0). Total playing area was rated on a scale from 0 (no playing area) to 10 (very large area).

#### 2.2.3. Background Information

Background information regarding the childcare centers, such as the number of enrolled children, was recorded during interviews with the manager of each childcare center. In addition, child's sex and age were assessed by asking present staff after the observations were finished. Weather conditions and outdoor temperature were recorded per observation day.

### 2.3. Statistical Analyses

All analyses were performed using SPSS 17.0. In all analyses *P* values <.05 were considered statistically significant. For activity level, the mean of the scores of both observers was calculated. Cohen's kappa was used to determine the interrater reliability (IRR) of the two observers. This measure indicates the proportional agreement between two observers, corrected for chance agreement [30]. The IRR was .7 (*P* < .001) in the current study, indicating substantial agreement [31].

All analyses were performed separately for indoor and outdoor observations. Various background characteristics were explored using descriptive statistics. The distribution of the physical activity facilities and the mean activity levels corresponding to the presence and absence of these facilities were explored. The significance of the differences between the activity levels with and without the facilities present were examined using *t*-tests. The association between total playing area and the physical activity level was examined using Pearson's correlations.

Next, we conducted backward regression analyses with the physical activity facilities and child characteristics (age and sex) as independent variables and the activity level as the outcome variable. Insignificant independent variables were stepwise deleted from the model in order of their significance, starting with the least significant variable. This procedure was repeated until all remaining independent variables were significant. 

Stepwise multilevel linear model analyses with 3 levels (i.e., measurement level; child level; center level) were executed to examine the association between activity level and the physical activity facilities, while modeling the interdependence between observations within individuals and between individuals within childcare centers. In the starting model, random intercepts at the child and center level were included, as well as a first-order autoregressive (AR(1)) correlation structure for repeated measures. Furthermore, random slopes at the centre level were included for child sex and age. Insignificant random effects were backward removed from the model, starting with the least significant random effect. When all remaining random effects were significant, the fixed effects were examined. The fixed effects were then examined analogous to the regression analyses described above (i.e., through the backward procedure).

We performed both the multivariate analyses where we corrected for the multilevel structure of data, and those without correction of the data, because the multilevel analyses might unintentionally overcorrect for the dependence between repeated measures within one subject in case an activity lasted longer than the duration of a single observation period (i.e., 45 seconds). This might especially be the case for various sedentary activities, such as playing in the sandbox.

## 3. Results

Five children were not present during the observations, leading to a total of 175 children that were each observed eight times. This resulted in 1400 observations per observer (i.e., 2800 single observations). Data regarding 18 observation periods were missing because these children were absent during one or more of the eight observation periods (e.g., because parents had already picked them up), which left 1382 observation periods for analyses. Eighty-nine (50.9%) of the observed children were male. The mean age was 2.6 years, with 75 two-year olds (42.9%) and 100 three-year olds (57.1%). The mean outdoor temperature during the observations was 20.4°C (range: 14°C–26°C). Most of the time the weather was sunny with clear skies (37.7%); least prevalent weather type was rain (11.4%).

### 3.1. Physical Activity Level

The mean activity intensity level during indoor observations was 2.36 (on a scale from 1 to 5). Only 5.5% of the indoor physical activity observations were classified as MVPA (moderate and vigorous physical activity; activity level ≥4), whereas 59.4% were classified as sedentary behavior (activity level ≤2). Outdoors, the mean activity level was 2.82, with 21.3% being MVPA, and 31.2% being sedentary.

### 3.2. Physical Activity Facilities

Table 1 provides an overview of the physical activity facilities in the 9 childcare centers. Indoors, all childcare centers provided balls and floor play equipment, and most (89%) also had portable and fixed climbing structures such as ladders and jungle gyms available. None of the centers had an indoor structured track, merry-go-round, tunnel, sandbox, or swinging equipment. The mean size of the indoor playing area was rated 6.11 (*sd*⁡ = 2.21) on a scale from 1 (no playing area) to 10 (very large playing area). 

In line with indoors, all childcare centers also provided balls outdoors. In addition, all centers had push or pull toys and riding toys available outdoors, as well as sand or water toys and balancing surfaces. Most centers (89%) also provided fixed climbing structures, fixed slides, and a sandbox outdoors. None of the observed childcare centers had a merry-go-round or twirling equipment available outdoors. The size of the outdoor playground was rated with an average of 6.22 (*sd*⁡ = 3.07).

### 3.3. Associations between Facilities and Physical Activity Level

#### 3.3.1. Indoor Facilities and Activity Level

Table 1 shows the mean activity levels corresponding to the indoor facilities being either present or not. Children were significantly more active indoors if jumping equipment, push or pull toys, portable slides, fixed slides or balancing surfaces were available indoors. They were significantly less active if sand or water toys were available indoors. The size of the indoor playing area was significantly positively correlated with children's activity levels (correlation coefficient *r* = .17, *P* < .001).

Table 2 shows the adjusted associations of the background factors and indoor physical activity facilities with indoor activity levels, with and without correction for the multilevel structure of the data (i.e., regression analyses and multilevel analyses, resp.). Both analyses show that riding toys are negatively associated with children's indoor activity levels, while the size of the playing area is positively associated with the activity levels.

#### 3.3.2. Outdoor Facilities and Activity Level

The mean activity levels of the children in the childcare centers where the various outdoor facilities were either present or not are presented in Table 1. The children were significantly more active in case floor play equipment, jumping equipment, a structured track, fixed climbing structures, fixed slides, tunnels, or a sandbox were available outdoors. None of the outdoor physical activity facilities was associated with a lower activity level in the bivariate analyses. The size of the outdoor playground was significantly positively correlated with children's activity level (*r* = .13, *P* = <.001). 

Table 3 shows the adjusted associations of the background factors and outdoor physical activity facilities with outdoor activity levels, with and without correction for the multilevel structure of the data. The analyses without correction for the multilevel structure of the data (the regression analyses) show that older children were significantly more active outdoors than younger children. Furthermore, portable jumping equipment and having a structured track showed a positive association with outdoor activity levels. Portable slides, sandboxes, and swinging equipment outdoors were negatively associated with children's activity level. However, in the multilevel analyses, only the presence of a structured track was still positively associated with children's activity levels.

## 4. Discussion

The current study examined the association between the availability of various physical activity facilities at the childcare center and 2- and 3-year olds' physical activity levels. In general, children's activity levels were comparable to those found in an earlier study applying the same protocol in US childcare centers (mean overall activity level 2.55 [22], compared to 2.59 in the current study). With regard to the associations with physical activity facilities, the study showed different results for indoor and outdoor observations. 

Indoors, children were more active when more space was available for playing. This is in line with a previous study by Cardon and colleagues [32], who found that the number of children per square meter was inversely correlated with activity levels, although this study regarded outdoor activity. We failed to find such an inverse association between the size of the playing area and activity levels outdoors. This could perhaps be attributed to the fact that Cardon et al. [32] took the number of children present also into account, which we did not. Also qualitative studies among childcare workers often mention space limitations as an important barrier for sufficient physical activity (e.g., [33, 34]).

In line with what could be expected from previous studies, which summarized the facilities assessed in the current study into one measure for activity opportunities and found a positive association between those activity opportunities and activity levels [21, 22], several outdoor physical activity facilities were positively associated with children's activity levels. Children were significantly more active when jumping equipment was present, as well as when a fixed track was marked on the playground. The latter is in line with an experimental study that showed that multicolor playground markings can increase children's activity levels, even in the long run [27].

However, there were also several play facilities that were found to be inversely associated with children's activity levels. Indoors the availability of riding toys seemed to decrease children's activity levels, and outdoors portable slides, sandboxes, and swinging equipment were associated with lower activity levels. With regard to the riding toys, this could probably be explained by space limitations and regulations indoors. The limited space restrains them from going very fast [33]. In addition, many childcare centers have safety policies or rules that limit vigorous physical activity such as running or riding very fast on a bike [33–36]. This results in the children merely sitting on a riding toy. Children in sandboxes are most of the time sitting, squatting, or standing, which explains the inverse association between sandboxes and activity level. With regard to slides and swings, we believe the negative association might stem from the fact that only one child can use these facilities at a time, and the other children are standing still, waiting for their turn. Moreover, once the child is using these equipments, he or she is again mainly sitting still. Many facilities that seem activity promoting at first glance thus appear to have the opposite effect on children's physical activity. 

The negative associations found between various facilities and children's activity levels make the use of “activity opportunity" sum scores such as the EPAO score [18] (of which we used the separate components in the current study) at least questionable: not all supposed activity promoting facilities seem to actually be activity-promoting. However, the differences between the bivariate analyses and the multivariate analyses demonstrate that one can also not take only one or a few of the facilities into account when looking at the influence of the physical childcare environment on children's activity levels. The sandbox is a good example of this. In the bivariate analyses, the presence of a sandbox at a childcare center was positively correlated with the activity levels of the children at that childcare center. However, the multivariate analyses showed a negative association between both. In practice, childcare centers that have a sandbox probably also have many other facilities available that do increase children's activity levels, because they have a larger budget for play facilities for instance, and it is actually a confounding effect that explains the positive bivariate association between the sandbox and activity levels. Indeed, secondary chi-square tests showed that the childcare centers that had a sandbox also significantly (*P* < .001) more often had various other physical activity facilities available, including jumping equipment and a structured track at the playground, which were both found to be positively associated with outdoor activity levels. In conclusion, studies examining influences of the physical childcare environment on children's activity level could probably best include a wide range of facilities, for instance, based on existing observation instruments such as the EPAO instrument [18], but refrain from summarizing these facilities into one measure for activity opportunities or childcare quality [20–26]. 

We found that the variability of the physical childcare environments was quite limited. Several facilities were provided in either all childcare centers (i.e., balls indoor and outdoor, indoor floor play equipment and outdoor push or pull toys, riding toys, sand or water toys and balancing surfaces) or in none of the centers (i.e., indoor structured track, merry-go-round, tunnels, sandboxes, and swinging equipment and outdoor merry-go-round or twirling equipment) in the current study. In case of the facilities that were present in none of the centers, this partly has to do with cultural differences between The Netherlands (in which the present study was conducted) and the USA (in which the EPAO instrument was developed [18]). Merry-go-rounds, for instance, are not common on playgrounds in The Netherlands. But the limited variability might also be partially due to the fact that all centers in our sample were part of the same coordinating organization, and adhere to central guidelines and policies of that organization. In that respect it would be interesting to repeat the current study in a broader sample of childcare centers. This does not solve the problem completely however, as part of the limited variability is probably not only a problem within our sample but in childcare centers in general. All childcare centers probably provide balls, for example. Experimental studies would be needed to further examine the influence of the different facilities on children's activity levels. 

A strong point of the current study is that the environment and physical activity intensity were directly observed and that it did not rely on less valid measures such as self-report. Furthermore, all observations were performed by 2 observers and interobserver reliability indicated substantial agreement [31]. However, although we based the choice for the length of the observation period (15 seconds) on the design of an earlier study using the same instruments [22], a longer or shorter observation period could also have been chosen, which would possibly have influenced the findings. Another point of consideration is whether the total observed time is representative for a child's activity during a whole day at childcare. However, observations were performed in each childcare centre during both a morning and an afternoon, which covers a regular day at childcare in The Netherlands (9:00 AM–5:00 PM). Finally, children's behavior could have been influenced by other variables that were not taken into account in this study. For instance, various social environmental factors such as prompts by peers and staff have been previously linked to children's physical activity level (e.g., [21]) but were not taken into account in the current study. 

## 5. Conclusions

Childcare organizations can use the findings of the current study to optimize the physical environment to promote children's physical activity. Not all childcare play facilities that were expected to be physical activity promoting were actually associated with increased activity levels in children. For promoting physical activity, childcare centers should try to optimize indoor space for playing, create outdoor play ground markings, and provide jumping equipment. The use of riding toys should probably be avoided in restricted spaces. Finally, the use of various larger facilities such as slides, swings, and sandboxes should be further examined, since they possibly limit children's activity levels. These findings need further testing, specifically using experimental research designs. 

With regard to future research into environmental influences on physical activity at childcare, the current study showed that physical activity facilities are probably better not summarized into general quality measures. It is, however, important to map the physical environment as completely as possible. Existing instruments (e.g., [18, 19]) are very useful for this purpose, although some cultural adaptations might be necessary, depending on the country in which the research is conducted.

## 6. Future Directions

The current study examined the influence of physical activity facilities on children's activity levels at childcare. Many other studies have previously examined the association between the childcare environment and children's activity levels, applying many different research designs and various focuses [16, 20–26, 32, 37–40]. However, these studies have in common that they mostly have a narrow, one-sided view on these environmental influences. This view mostly incorporates a physical activity stimulating environment as a whole, without further specifying contributes of individual environmental characteristics. Ecological models integrate environmental influences with individual influences and influences from other settings (e.g., the home environment) [41, 42], giving an overview of the broader picture in which the child's behavior takes place. We feel that also in the case of childcare, future studies should not only examine the direct influences of the childcare environment, but also the interactive influences of the childcare environment with children's characteristics (e.g., sex, temperament) and the home environment.

## Figures and Tables

**Table 1:** Physical activity facilities at childcare centers (*N* = 9) and corresponding activity levels children (*N* = 175).

Equipment		Indoor (Obs. = 710)				Outdoor (Obs. = 689)			
		Not present		Present		Not present		Present	
		Frequency	Mean activity level (sd)^a^	Frequency	Mean activity level (sd)^a^	Frequency	Mean activity level (sd)^a^	Frequency	Mean activity level (sd)^a^
	Balls	0	—	9	2.36 (.61)	0	—	9	2.82 (.72)
	Climbing structures	1	2.36 (.68)	8	2.36 (.60)	7	2.80 (.74)	2	2.87 (.64)
	Floor play equipment	0	—	9	2.36 (.61)	7	2.79 (.72)	2	2.96 (.69)^∗^
	Jumping equipment	4	2.26 (.49)	5	2.40 (.85)^∗∗^	4	2.71 (.71)	5	2.93 (.71)^∗∗^
Portable	Push/pull toys	6	2.29 (.55)	3	2.44 (.66)^∗∗^	0	—	9	2.82 (.72)
	Riding toys	7	2.39 (.63)	2	2.30 (.57)	0	—	9	2.82 (.72)
	Slides	8	2.29 (.55)	1	2.70 (.76)^∗∗^	6	2.80 (.71)	3	2.89 (.76)
	Sand/water toys	4	2.45 (.67)	5	2.29 (.55)^∗∗^	0	—	9	2.82 (.72)
	Twirling equipment	4	2.32 (.59)	5	2.38 (.62)	9	2.82 (.72)	0	—

	Structured track	9	2.36 (.61)	0	—	3	2.71 (.71)	6	2.93 (.71)^∗∗^
	Merry-go-round	9	2.36 (.61)	0	—	9	2.82 (.72)	0	—
	Climbing structures	1	2.29 (.40)	8	2.36 (.62)	1	2.67 (.69)	8	2.86 (.72)^∗∗^
	See-saw	8	2.36 (.62)	1	2.34 (.51)	5	2.79 (.72)	4	2.87 (.72)
Fixed	Slides	8	2.29 (.55)	1	2.70 (.76)^∗∗^	1	2.67 (.69)	8	2.86 (.72)^∗∗^
	Tunnels	9	2.36 (.61)	0	—	5	2.76 (.70)	4	2.94 (.74)^∗∗^
	Balancing surfaces	2	2.18 (.49)	7	2.38 (.63)^∗∗^	0	—	9	2.82 (.72)
	Sandbox	9	2.36 (.61)	0	—	1	2.67 (.69)	8	2.86 (.72)^∗∗^
	Swinging equipment	9	2.36 (.61)	0	—	6	2.84 (.73)	3	2.78 (.70)

Obs.: number of observations.

^∗^ *P* < .05, ^∗∗^ *P* < .01.

^a^Activity level assessed on a scale from 1 to 5. Significance levels of *t*-tests comparing the activity levels in centers with the equipment being present or not.

**Table 2:** Backward regression analyses and multilevel analyses of the association between the indoor childcare physical activity facilities and children's physical activity levels (number of observations = 710).

	Regression analysis^a^		Multilevel analysis^a,b^	
	Regression coefficient (B)	*P* value	Regression coefficient (B)	*P* value
Portable riding toys	−.32	<.001	−.31	<.001
0 = not present, 1 = present				
Size playground	.10	<.001	.10	<.001
0 (very small)–10 (very large)				

^a^Variables excluded from the analyses because they were present in either all or none of the childcare centers: portable equipment: ball equipment and floor play equipment; fixed equipment: structured track, merry-go-round, tunnels, sandbox, and swinging equipment. Variables excluded from the final model because they were nonsignificant: portable equipment: climbing structures, jumping equipment, push/pull toys, slides, sand/water toys, and twirling equipment; fixed equipment: climbing structures, see-saw, slides, and balancing surfaces; background variables: sex, age.

^b^Final model comprises a first-order autoregressive (AR1) correlation for repeated measures within children. Other random effects were not included in the final model.

**Table 3:** Backward regression analyses and multilevel analyses of the association between the outdoor childcare physical activity facilities and children's physical activity levels (number of observations = 689).

	Regression analysis^a,b^		Multilevel analysis^a,c^	
	Regression coefficient (B)	*P* value	Regression coefficient (B)	*P* value
Age in years	.13	.019	—	—
Portable jumping equipment	.36	<.001	—	—
0 = not present, 1 = present				
Portable slides	−.55	<.001	—	—
0 = not present, 1 = present				
Fixed structured track	.53	<.001	.23	.003
0 = not present, 1 = present				
Fixed sandbox	−.49	.002	—	—
0 = not present, 1 = present				
Fixed swinging equipment	−.41	.001	—	—
0 = not present, 1 = present				

^a^Variables excluded from the analyses because they were present in either all or none of the childcare centers: portable equipment: ball equipment, push/pull toys and riding toys, and sand/water toys, twirling equipment; fixed equipment: merry-go-round, balancing surfaces.

^b^Variables excluded from the final model because they were nonsignificant: portable equipment: climbing structures, floor play equipment; fixed equipment: climbing structures, see-saw, slides, and tunnels; background variables: sex.

^c^Final model comprises a first-order autoregressive (AR1) correlation for repeated measures within children. Other random effects were not included in the final model. Variables excluded from the final model because they were non-significant: portable equipment: climbing structures, floor play equipment, jumping equipment, and slides; fixed equipment: climbing structures, see-saw, slides, tunnels, sandbox, and swinging equipment; background variables: sex, age.
